# Extensive Epidermal Skin Loss Secondary to HSV Type One: Neonatal Management Challenges

**DOI:** 10.1155/2019/2459219

**Published:** 2019-12-05

**Authors:** Rebecca J Calthorpe, Emma Spencer, Jane C Ravenscroft, Ting S Tang, Anna E Martinez, Anjum Deorukhkar

**Affiliations:** ^1^Department of Neonatology, Nottingham University Hospitals NHS Trust, Nottingham, UK; ^2^Department of Dermatology, Nottingham University Hospitals NHS Trust, Nottingham, UK; ^3^Department of Dermatology, Great Ormond Street Hospitals, London, UK

## Abstract

We describe a rare case of a preterm neonate presenting at birth with extensive epidermal skin loss of over 90% due to disseminated herpes simplex virus type one infection. Differential diagnosis included aplasia cutis and epidermolysis bullosa. Serum PCR and mouth swabs confirmed HSV type one, and the patient required three weeks of treatment with intravenous aciclovir, followed by oral aciclovir. We describe the management challenges and give practical solutions applicable to the care of a neonate presenting with widespread skin loss due to any aetiology.

## 1. Introduction

This report describes a rare case where a thirty-week gestation neonate had over 90% epidermal skin loss at delivery due to disseminated, transplacentally acquired herpes simplex virus (HSV) type one. This resulted in complex neonatal care that was atypical to a baby of this gestation. Typically, the neonatal course for an infant born at this gestation in the United Kingdom (UK) would involve a short period of respiratory support and establishment of central venous access to administer parenteral nutrition until feeds are introduced. They would receive a course of antibiotics, may require phototherapy to manage jaundice, and will routinely need cranial ultrasound scans and screening for retinopathy of prematurity. Survival rates in the UK for a thirty-week gestation neonate are 96% [1]. This markedly contrasts survival rates in paediatric burn patients where those with 90% skin damage have 50% mortality at one year after injury [2]. Consequently, the extent of skin loss experienced in this case required a tailored management approach.

In July 2019, the British Paediatric Surveillance Unit (BPSU) launched a two-year study that aims to assess the treatment burden of HSV in neonates less than 90 days, in order to inform detection and management decisions [3]. This will be beneficial in the treatment of neonatal HSV in the future, particularly as the disease has devastating consequences. Currently, there is limited literature available to guide the management of widespread skin loss due to HSV. Therefore, given the success of this case despite its complexity, we provide information on the diagnostic challenges and the individualised care employed. We also give practical treatment strategies for the management of a neonate with widespread skin loss due to HSV that are applicable to any aetiology of widespread epidermal skin loss in a neonate (Table 1).

## 2. Case Description

**Figure 1:**
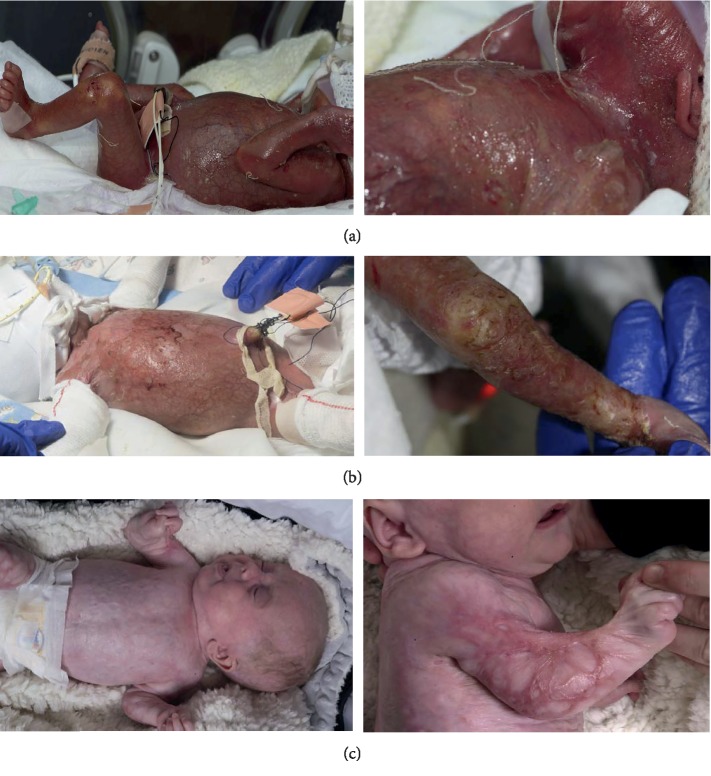
Images of the neonate following presentation at birth (a), at one week of age with some visible skin reepithelisation (b), and at four months of age following the development of cribiform scarring and contractures (c).

A thirty-week gestation preterm neonate (birth weight: 1250 g) was born via normal vaginal delivery to a healthy primip mother in a hospital where there was tertiary level neonatal care. There had been spontaneous rupture of membranes 48 hours prior to delivery, and a background history of a small antepartum haemorrhage at twenty-nine weeks. Prior to this, the pregnancy had been uncomplicated with a normal antenatal scan performed at twenty weeks gestation. There was a known maternal history of Group B Streptococcal infection and a past history of oral herpes simplex infection in both parents but no known genital lesions. At delivery, the infant had extensive epidermal skin loss of over 90% with sparing of the soles of the feet, fingers, genital area, and central face (Figure 1(a)). The extent of skin loss was estimated using the Mersey Burns calculator [4]. Widespread skin loss resulted in complex care beyond that usually required for an infant born at this gestation.

### 2.1. Respiratory

He was intubated and given 200 mg/kg surfactant in the delivery suite due to poor respiratory drive. He subsequently required minimal invasive ventilation; however, extubation to noninvasive support was impeded by concerns that the CPAP face mask would exacerbate skin damage. He was also on high-dose opiates for pain management with the associated risk of respiratory depression. He therefore remained intubated until day seven of life, at which point he was extubated straight into head box oxygen.

### 2.2. Monitoring and Intravenous Access

A fundamental issue in his care was that intravenous access was difficult to obtain and secure. Extensive skin loss also hindered monitoring of observations, normally done via blood pressure cuffs, saturation probes, and electrocardiogram leads. Heart rate and blood pressure were monitored invasively via an umbilical arterial catheter (UAC), which avoided further skin damage and facilitated blood sampling. Unfortunately, the umbilical venous catheter (UVC) was not optimally placed or secured, hence required reinsertion on day three and again on day seven to attempt better placement. Long-line insertion in the limbs was not feasible; consequently the umbilical venous catheter was used for an extended period beyond what was considered as acceptable routine practice on the neonatal unit. A long line was later inserted in a scalp vein due to relative skin sparing in this area. In total, the UVC and UAC remained in situ for ten days. During this time, routine monitoring included twice daily examination of the umbilicus and hourly documentation of the fluid infusion volume and pressure. The UVC tip from the replaced umbilical venous catheters was sent for microbiology to assess the likelihood of possible secondary central line infection (Table 2).

### 2.3. Fluid and Nutrition

He required an initial 40 ml/kg fluid bolus and rapid escalation of fluids to a maximum of 200 ml/kg/day to account for high insensible fluid loss from the skin, associated hypotension, and to avoid acute prerenal injury. He was also recurrently anaemic and thrombocytopenic. Local hospital guidelines detailed the transfusion thresholds for both red cell and platelet transfusions [5, 6]. A transfusion threshold of haemoglobin below 120 g/l for a ventilated preterm and platelets <30 × 10 9/L for a sick preterm were employed and administered at a volume of 20 ml/kg and 10 ml/kg, respectively. Parenteral nutrition was administered via the suboptimally placed umbilical venous catheter, and trophic feeds of expressed breast milk were commenced at the earliest opportunity. He remained on trophic feeds for nine days due to the risk of necrotising enterocolitis in a preterm with umbilical lines in situ and on high-dose opiates. Feeds were increased quickly from day nine, and full feeds at 165 ml/kg/day of expressed breast milk were established by day twelve.

### 2.4. Jaundice and Phototherapy

Within the first twenty-four hours, serum bilirubin was measured on the exchange transfusion line on the National Institute for Health and Care Excellence (NICE) treatment threshold graphs. The efficacy of phototherapy in a neonate with extensive skin loss was unknown, but despite this conventional blue light phototherapy was employed at an intensified level using three separate overhead lights due to the risk of kernicterus. Once the serum bilirubin was below the exchange transfusion line, the use of phototherapy was moderated due to concerns that it would exacerbate skin damage and insensible fluid loss. A pragmatic approach was taken with six hourly serum bilirubin measurements used to aid decisions on the duration of phototherapy. Phototherapy was stopped by day two, when the bilirubin level reached the phototherapy treatment line. Overall, he had a good response to treatment with no obvious skin exacerbation.

### 2.5. Pain

This was one of the most challenging aspects of care. Pain was assessed hourly using the Pain Assessment Tool (PAT), which uses a combination of physiological observations, behavioural parameters, and nursing perception to give a score out of twenty for pain [7]. Due to consistently high pain scores, exacerbated by spontaneous movement and handling, he required rapid escalation of analgesia. The analgesia prescribed was that typically administered in the paediatric intensive care setting. Use of ketamine and fentanyl infusions up to a maximum dose of 100 micrograms/kg and 5 micrograms/kg, respectively, helped achieve better pain control. Additional intravenous boluses were administered before routine cares and during dressing changes.

### 2.6. Microbiology

Serum PCR and mouth and skin swabs taken on admission to the neonatal unit were positive for herpes simplex virus type one and remained positive until day nineteen of life (Table 2). There is a high risk of neurological complications, including meningitis and encephalitis in HSV infection. Lumbar puncture could not be performed due to extensive skin loss on the back. Instead, close monitoring of viral load based on serum PCR was performed on advice of Microbiology. Twenty-one days of intravenous aciclovir was completed, followed by a six-month course of oral aciclovir at a dose of 300 mg/m^2^/day. An added complication from day three was infection with Enterococcus grown from the UVC tip and blood cultures (Table 2); this was successfully treated with a seven-day course of intravenous vancomycin.

### 2.7. Neurology

Mild to moderate ventriculomegaly was present on serial cranial ultrasound scans, likely posthaemorrhagic in origin. Subsequent MRI brain performed at corrected term gestation was normal.

### 2.8. Ophthalmology

Routine screening for retinopathy of prematurity was performed in view of multiple risk factors, and this was normal at the time of discharge from hospital at 37 + 5 weeks corrected gestation.

### 2.9. Multidisciplinary Team (MDT)

There was extensive MDT involvement throughout admission. His presentation was not classical for one condition. The national epidermolysis bullosa (EB) team based at Great Ormond Street Hospital gave immediate telephone support, followed by an outreach service from Birmingham, who were geographically closer. They performed a skin biopsy, provided dressings advice, and supported the family during the initial diagnostic period. Severe subtypes of epidermolysis bullosa were excluded with the skin biopsy, and on day seven he was noted to have reepithelisation of skin (Figure 1(b)). Dermatology supported with diagnostic and treatment advice, in addition to liaising with the Plastics team on skin dressings. His skin was managed with dressings, emollients, and Octenisan wash (Table 1). Both teams played a role in the education of the neonatal team and parents around skin treatment. Once the skin started to reepithelise, he developed contractures at multiple joints with widespread cribriform scarring. This was managed by Plastics and Physiotherapy who promoted mobilisation and the application of splints. The success of this case is owed to the support of the dedicated MDT in a tertiary centre with close involvement of the EB team.

### 2.10. Long Term

At 34 weeks corrected gestation, he was transferred to the local neonatal unit for ongoing care. He was subsequently discharged home at 37 + 5 weeks corrected gestation with a plan for long-term follow-up with a general paediatrician. At this point, there were no further concerns with acute infection and he continued on the planned six-month course of oral aciclovir. His skin was managed with regular application of Dermol and Paraffin lotion on advice of the local Dermatology team. At the time of submitting this case report for publication, he was four months of age and the main ongoing issue was related to extensive cribriform scarring and skin contractures (Figure 1(c)). This was being managed by outpatient Physiotherapy and the Plastics team who were considering the need for skin grafting long term.

## 3. Discussion

Congenital HSV infection has an estimated incidence from 1 : 1400 to 1 : 30000 deliveries [8]. It can result in long-term neurodisability or death with a mortality rate of 50% [8]. Over 75% of cases are caused by HSV type two and are acquired peripartum. This case of transplacentally acquired intrauterine infection with HSV type one is extremely rare. Intrauterine HSV1 infection has an increased risk of preterm labour, as seen in this case, and intrauterine growth restriction. There are three well-described presentations of HSV infection: disseminated disease (20%); central nervous system disease (30%); and skin, eyes, and mouth disease (45%) [8]. Cutaneous lesions are the most common presentation in up to two thirds of affected neonates, either in isolation or in association with central nervous system disease or disseminated disease [8, 9]. Types of cutaneous presentations include vesicles, bullous lesions, erosions, ulcers, pustules, erythema, plaques, and hypopigmented scarring. To our knowledge, widespread extensive skin loss, as seen in this case, has not been reported in the literature [9].

The differential diagnosis at presentation included epidermolysis bullosa, aplasia cutis, or skin loss secondary to infection. Assessment of associated congenital abnormalities may give a clue to a genetic diagnosis; however, there should be a high clinical suspicion of HSV and early empirical treatment with intravenous aciclovir. Viral and bacterial skin swabs and serum samples should be taken for PCR prior to commencing antimicrobial treatment. There are two national Paediatric EB services in the United Kingdom (London and Birmingham) who can be contacted regarding skin care management and will give advice about any child with skin fragility. They also have written clinical guidelines detailing skin care, which were employed in the management of this neonate [10].

Given the rarity of this presentation and the difficulties in management, this case report has provided practical solutions for the additional treatment challenges faced when caring for a neonate with extensive skin loss of any aetiology (Table 1). Early recognition, prompt referral to the national Paediatric EB team, and tertiary neonatal intensive care are essential. MDT involvement is paramount and can be credited with the successful management of this unusual presentation.

## Figures and Tables

**Table 1:** Table outlining the key management points when treating a neonate with widespread skin loss.

System-based considerations when managing a neonate with widespread skin loss	
Respiratory	Invasive ventilation can allow the clinician to optimise analgesia administration and avoid further skin damage to the head and face.

Intravenous access	Intravenous access may be difficult to obtain and secure; maximise the duration of central line access with careful monitoring. Umbilical access should be secured to the umbilical cord and not the skin. UAC access facilitates blood sampling as heel prick blood sampling should be avoided.

Monitoring	Tailor how routine observations are performed and do not attach electrodes to the skin or use skin temperature probes. An oxygen saturation probe can be used to monitor the heart rate, and blood pressure measurements can be obtained via a UAC.

Fluids	There will be excessive fluid loss via the skin; therefore, fluid balance monitoring should be documented hourly to aid decisions on fluid management.

Nutrition	Nutrition is important to promote skin healing and should be considered early. If central venous access has been obtained, parenteral nutrition can be prescribed until milk feeds are commenced.

Analgesia	Hourly monitoring of pain using a classified pain scoring system will direct pain management. Rapid escalation of analgesia with drugs not typically prescribed in neonates may be needed; therefore, expert pharmacy advice should be accessed where available.

Jaundice	In this case, phototherapy was implemented effectively to treat jaundice with no obvious exacerbation to the skin.

Neurology	The clinician should be aware of the neurological complications related to HSV infection. If a lumbar puncture cannot be performed, liaise with a virologist to discuss treatment options. Cranial ultrasound and MRI scans should be used to aid diagnosis of neurological morbidity.

Microbiology	Consider HSV infection in a neonate presenting with widespread skin loss and obtain samples for viral PCR. Administer intravenous aciclovir early. There is an increased risk of infection; therefore, have a low threshold for commencing antimicrobial treatment if there are signs of infection.

Place of care	Transfer to a tertiary neonatal unit is advisable where all members of the MDT are available to offer expert review and treatment input.

Communication	Provide clear communication with the family at all stages of admission about treatment decisions and outcome expectations.

Specific skin considerations when managing a neonate with widespread skin loss	

Handling	Ensure careful handling to avoid skin friction; handling techniques can be taught by the EB team.

Dressings	Give adequate analgesia 15 to 30 minutes prior to dressing application. Apply dressings to areas of raw skin, in this case Mepilex Transfer was applied, held in place with Tubifast bandage. Cling film can be used if no other dressings are immediately available.

Emollients	Apply emollients such as Dermol 500 and 50 : 50 Paraffin lotion to the skin. Emollients such as 50 : 50 Paraffin lotion can be applied to the fingers prior to handling.

UAC: umbilical arterial catheter; PCR: polymerase chain reaction.

**Table 2:** Table outlining the results of key microbiology results according to the day the test was taken and organised by the type of microbiological investigation.

Day	Microbiological investigation	Result
0	Blood PCR	HSV1
9	Blood PCR	Negative
16	Blood PCR	Low levels of HSV1
19	Blood PCR	Negative
24	Blood PCR	Negative

0	Skin swab	HSV1
16	Skin swab	Negative

0	Blood culture	Negative
4	Blood culture	Enterococcus
13	Blood culture	Negative

3	UVC tip	Enterococcus
7	UVC tip	Enterococcus
10	UVC tip	Enterococcus
10	UAC tip	Enterococcus

## Acronyms

ECG: Electrocardiogram
EB: Epidermolysis bullosa
HSV1: Herpes simplex virus type one
MDT: Multidisciplinary team
NICE: National Institute for Health and Care Excellence
PN: Parenteral nutrition
PCR: Polymerase chain reaction
UAC: Umbilical arterial catheter
UVC: Umbilical venous catheter.
